# Mutations and insights into the molecular mechanisms of resistance of *Mycobacterium tuberculosis* to first-line

**DOI:** 10.1590/1678-4685-GMB-2022-0261

**Published:** 2023-01-23

**Authors:** Nicolas de Oliveira Rossini, Marcio Vinicius Bertacine Dias

**Affiliations:** 1 Universidade de São Paulo, Instituto de Ciências Biomédicas, Departamento de Microbiologia, São Paulo, SP, Brazil. Universidade de São Paulo Instituto de Ciências Biomédicas Departamento de Microbiologia São Paulo SP Brazil; 2 University of Warwick, Department of Chemistry, Coventry, United Kingdom. University of Warwick Department of Chemistry Coventry United Kingdom

**Keywords:** Mycobacterium tuberculosis, antimicrobial resistance, isoniazid, pyrazinamide, ethambutol, rifampicin

## Abstract

Genetically antimicrobial resistance in *Mycobacterium tuberculosis* is currently one of the most important aspects of tuberculosis, considering that there are emerging resistant strains for almost every known drug used for its treatment. There are multiple antimicrobials used for tuberculosis treatment, and the most effective ones are the first-line drugs, which include isoniazid, pyrazinamide, rifampicin, and ethambutol. In this context, understanding the mechanisms of action and resistance of these molecules is essential for proposing new therapies and strategies of treatment. Additionally, understanding how and where mutations arise conferring a resistance profile to the bacteria and their effect on bacterial metabolism is an important requisite to be taken in producing safer and less susceptible drugs to the emergence of resistance. In this review, we summarize the most recent literature regarding novel mutations reported between 2017 and 2022 and the advances in the molecular mechanisms of action and resistance against first-line drugs used in tuberculosis treatment, highlighting recent findings in pyrazinamide resistance involving PanD and, additionally, resistance-conferring mutations for novel drugs such as bedaquiline, pretomanid, delamanid and linezolid.

Introduction

Tuberculosis (TB) is an infectious disease caused predominantly by *Mycobacterium tuberculosis* (Mtb). This disease is a serious problem for public health since it afflicted about 10 million people worldwide, which culminated in 1.3 million deaths only in 2020 This makes TB the second most common cause of death by a single infectious agent, only surpassed in recent years by COVID-19. Among the most used medicines in the treatment of TB, isoniazid (INH), pyrazinamide (PZA), ethambutol (EMB), and rifampicin (RIF) are called first-line drugs (Figure 1). These drugs are the first choice of treatment for TB, which has a regimen of about six months with co-administration of all of them in the first four months and two of them in the last two ones (World Health Organization, 2021). 

**Figure 1 -:**
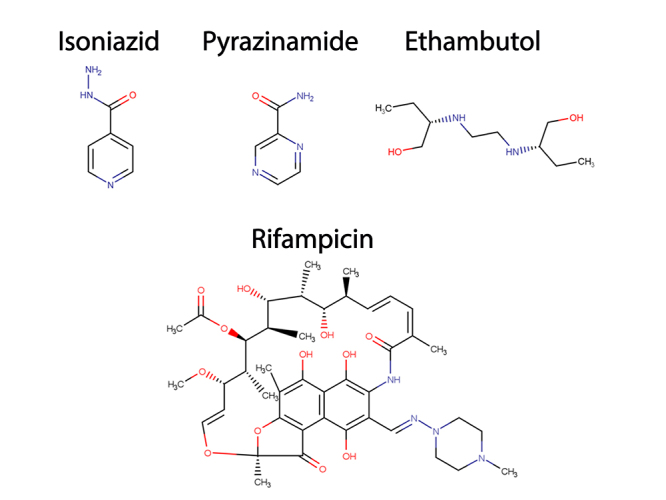
First-line drugs used in tuberculosis treatment. Structures for first-line drugs used in tuberculosis treatment, Isoniazid, Pyrazinamide, Rifampicin and Ethambutol. Structures were drawn using Marvin software (Cherinka et al., 2019).

Additionally, to the first-line drugs, other antimicrobials, including ethionamide (ETH), injectable aminoglycosides, fluoroquinolones, diarylquinolines, and nitroimidazoles can also be used, but only against resistant strains. These antimicrobials are denominated as second-line drugs (Figure 2).The second-line drugs have been proven to have lower efficacy and higher toxicity compared to first-line drugs and require a longer regimen of treatment that could take more than a year (Wolff and Nguyen, 2012; Gopal and Dick, 2014; World Health Organization, 2021). Nevertheless, new regimens, based on novel or repurposed drugs with anti-TB activity such as bedaquiline, delamanid, pretomanid, linezolid, clofazimine and moxifloxacin are currently in phase III clinical trials, aiming to reduce or simplify the current chemotherapy for MDR-TB and XDR-TB some of which are ZeNix, endTB and SimpliciTB. (Perrin et al., 2022).

**Figure 2 -:**
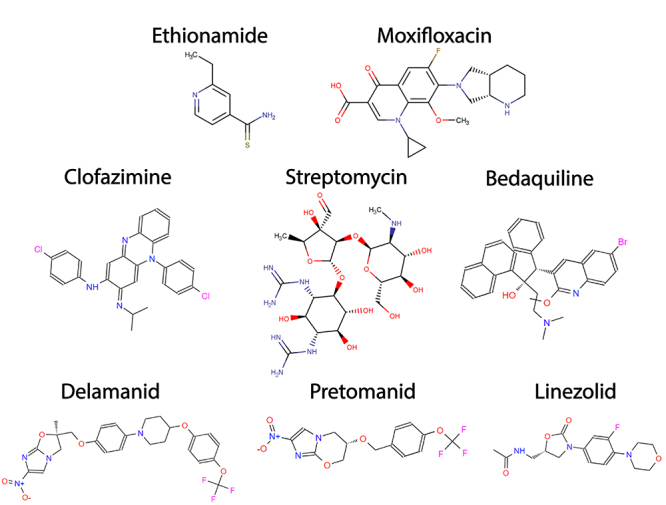
Second-line drugs used in tuberculosis treatment. Structures for some second-line drugs used in tuberculosis treatment, Ethionamide, Moxifloxacin (Fluoroquinolone), Clofazimine, Streptomycin (injectable aminoglycoside), Bedaquiline, Delamanid, Pretomanid and Linezolid Structures were drawn using Marvin software (Cherinka et al., 2019).

Worrisomely, because of the long treatment, which includes severe side effects that contribute to the non-effective adhesion of the regimen, the number of genetically resistant and multiresistant strains to all in-use drugs against TB is alarmingly high and increases every year. The resistance to antimicrobials in TB is predominantly caused by either intrinsic resistance (particularly because of the complex mycobacterial cell wall and the presence of a chromosomal β-lactamase) or by mutations in genes (including promotor and encoding regions) from the antimicrobials targets and/or key enzymes for activating pro-drugs, such as INH and PZA. Plasmid horizontal transference is not reported so far in Mtb and consequently, this is not considered an important aspect of mycobacteria antimicrobial resistance (Smith et al., 2012; Zhang and Yew, 2015). Mono resistance is common for INH, RIF and even streptomycin, however, many resistant strains of Mtb are resistant to at least two drugs. Based on that, the resistance in TB can be classified into multiresistant strains (MDR-TB), which consists of those strains that are resistant at least to INH and RIF; Pre-extensively drug-resistant TB (pre-XDR-TB), which includes those strains resistant to INH, RIF, a fluoroquinolone and a further injectable second-line drug, such as aminoglycosides; and extensive resistant strains (XDR-TB), which carry on all MDR-TB resistances and further resistances to at least one drug from the fluoroquinolone group combined with resistance to a group A drug, such as bedaquiline, levofloxacin, moxifloxacin or linezolid (World Health Organization, 2021). 

In this review, we highlight the genetic mechanisms of resistance identified in Mtb for the first-line drugs, including INH, PZA, RIF and EMB. Additionally, although this is not the main focus of this revision, we also succinctly discuss the mechanisms of action and resistance involved against the most important second-line drugs, including bedaquiline, pretomanid, linezolid and clofazimine. We discuss the findings aiming to understand the mechanisms of resistance in this bacteria, as well as the recently reported polymorphisms and resistance-conferring mutations described in the last 5 years for first-line drugs used in the active TB treatment. This gathering of information has a pivotal role in proposing new strategies for more personalized treatment and improving clinical practices contributing to avoiding the dissemination of MDR, Pre-XDR, and XDR strains.

## Resistance to INH

Isoniazid or isonicotinic acid hydrazide (INH) (Figure 1) is one of the most efficient anti-TB drugs (World Health Organization, 2021) and has been used as an anti-tubercular agent since 1952 (Selikoff et al., 1952). INH is formed by a hydrazine group attached to a pyridine moiety and is considered a narrow-spectrum antimicrobial with bactericidal activity against Mtb during the actively-growing bacterial phase and bacteriostatic against slow-growing and latent stage (Fernandes *et al*., 2017). INH is largely used against TB and it is one of the antimicrobials used in the standard TB regimen treatment (Sotgiu et al., 2015; World Health Organization, 2021). INH was first identified as a groundbreaking anti-TB agent in 1951, and since then has been widely used against TB (Murray et al., 2015). Because of that, it is not a surprise that INH resistance is identified in more than 11% of all TB cases (World Health Organization, 2021). 

**Figure 3 -:**
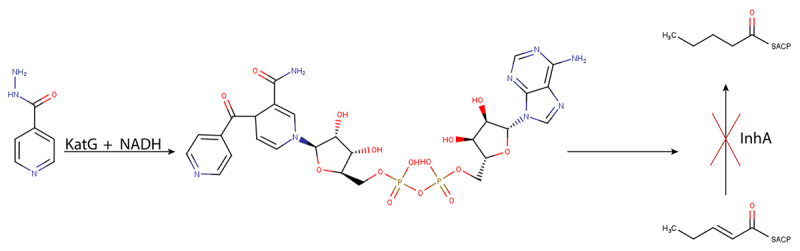
InhA inhibition by INH-NAD adduct. Mechanism of action of InhA and inhibition by INH. KatG activates INH to produce the adduct INH-NAD adduct, which is formed through a reaction with NADH. InhA is inhibited by INH-NAD adduct, which blocks the fatty acid elongation catalyzed by the FAS-II system. Adapted from Vilchèze and Jacobs 2007.

**Figure 4 -:**
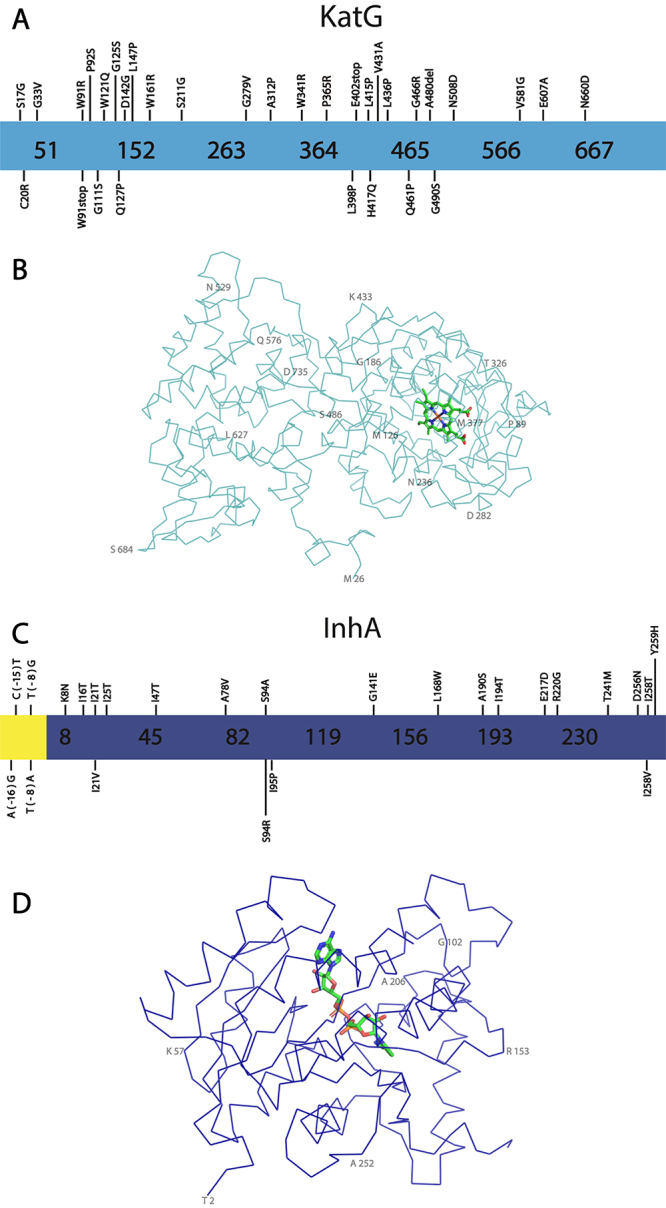
Structure and key mutations for KatG and InhA, proteins involved in INH resistance. **A -** Representation of KatG primary sequence is shown light blue, showing novel mutations. **B -** Representation of KatG structure. The contours of Cα are shown in light blue, while the Heme group is shown with carbon atoms in light green. Some residues are numbered in 50% opacity for better visualization. The KatG structure was obtained from PDB entry 2CCA (Zhao et al., 2006) and visualized using the software PyMOL (Schrödinger, 2015). **C -** Representation of InhA sequence in dark blue, showing the most significant mutations. The InhA promoter region is shown in yellow. **D -** Representation of the structure. The contours of Cα are shown in dark blue while the NADH is shown with carbon atoms in light green. Some residues are numbered in 50% opacity for better visualization. The structure was obtained from PDB entry 4TRN (Chollet et al., 2015) and the figure was prepared using the software PyMOL (Schrödinger, 2015)**.**

INH is a pro-drug and needs to be activated by a catalase-peroxidase system, particularly by the enzyme KatG (Zhang et al., 1992). KatG oxidizes INH in two steps: during the first one occurs the formation of isonicotinoyl radical; and in the second one, the radical reacts with ammonia to form isonicotinamide, the active state of INH (Bodiguel et al., 2001; Pierattelli et al., 2004; Timmins et al., 2004) (Figure 3). In addition to the active form of INH, several reactive oxygen species are also produced during this conversion (Shoeb et al., 1985). When activated, INH forms an adduct with NADH, the INH-NAD, through the formation of a covalent bond with the nicotinamide group of this coenzyme (Figure 3). INH-NAD is the responsible molecule for inhibiting the biosynthesis of mycolic acids in Mtb by binding to 2-*trans*-enoyl-acyl carrier protein reductase (InhA). This enzyme has a Rossmanoid fold and belongs to the NADH-dependent short dehydrogenase/reductase family and catalyzes the reduction of *trans-*2-enoyl-ACP fatty acids (Dessen *et al*., 1998) (Figure 4D). As an enzyme from the fatty acid system II (FAS-II), InhA is particularly involved in the elongation steeps, with the sequential extension of C_15_-C_18_ leading to the production of the long C_56_ fatty acid chains, which are precursors of mycolic acids (Marrakchi et al., 2000; Rawat et al., 2003; Massengo-Tiassé and Cronan, 2009; Zhu et al., 2013; Vögeli et al., 2018). At the InhA active site, INH-NAD competes with NADH inhibiting its activity and consequently causing the accumulation of saturated C_26_ fatty acids and stopping the production of mycolic acids, which are key components of the mycobacterial cell wall, contributing to the bacteria lysis (Wilming and Johnsson, 1999; Rawat *et al*., 2003). 

The INH mechanism of resistance is complex and not completely understood and maybe involves many genes, although reported mutations are predominantly identified on *katG* and *inhA* genes (Zhang et al., 1992). The mutations observed in *inhA* and *KatG* are mostly single missense point substitutions rather than deletions. However, deleterious mutations can also be found on *katG* (Heym et al., 1999) since this gene is a non-essential for Mtb survival. Indeed, the disruption of this gene brings an adaptative advantage under INH treatment and consequently contributes to spreading the INH resistance (Wengenack et al., 2004). 

KatG is a homodimeric enzyme, in which each monomer has 2 domains. These domains have a similar folding to other proteins from the peroxidase family, which are predominantly formed by α-helices (Bertrand *et al*., 2004). Although, both C-terminal and N-terminal domains are similar, the N-terminal domain binds a heme porphyrin, which is also part of the KatG active site, and the binding of INH closer to the heme-binding site was shown to be a key prerequisite for INH activation (Figure 4B) (Zhao et al., 2006).

In terms of INH resistance, the most common substitutions on KatG occur in the active site, particularly at the INH binding site, which includes the residues R104, H108 and S315. Additionally, several substitutions are also observed in residues involved in the heme binding site (Figure 4B), such as H270 and T275 (Ramaswamy and Musser, 1998). Mutations in these residues alter substrate affinity, or change the accessibility to the heme group, leading to lower catalase-peroxidase activity, and causing inefficiency in the INH activation (Carpena et al., 2003; Bertrand *et al*., 2004). 

InhA mutations also represent an important factor for INH resistance. One of the most common mechanisms of INH resistance involves the overexpression of *inhA* gene, caused mainly by mutations in the promoter region (Musser et al., 1996; Ramaswamy and Musser, 1998; Vilchèze et al., 2006). Alternatively, several mutants were also identified to have substitutions on the coding region of *inhA* leading to missense mutations. Most of the mutant enzymes that were biochemically or biophysically characterized so far indicate a decrease in the NADH binding affinity to InhA and consequently to INH-NAD, increasing the turnover of the coenzyme and adduct in the protein active site, which favors the enzymatic catalysis (Banerjee et al., 1994; Whitney and Wainberg, 2002). 

A review published by Unissa et al. (2016) describes in detail several KatG and InhA mutations discovered up to 2016, and from those, the most clinically relevant are briefly reported herein, along with more recently described mutations (Unissa *et al*., 2016).

The S315T mutation in KatG is one of the most predominant in INH resistant (INH^R^) Mtb strains. This substitution leads to the narrowing of the access pathway to the heme group from 6Å to 4.7Å, which decreases the binding affinity for INH and reduces the INH activation and NAD-INH adduct formation. Interestingly, this substitution partially maintains the KatG catalase-peroxidase function (Yu et al., 2003; Ghiladi et al., 2004; Zhao et al., 2006). 

On the other hand, His108 is also reported to be a residue involved in INH binding. Two substitutions that have been identified include H108E and H108Q. These two mutations also decrease the affinity of KatG to INH, probably because of weaker interaction and charge repulsion with the substituted residue to the INH hydrazinyl group, which disturbs the INH activation pathway (Musser et al., 1996; Bertrand *et al*., 2004). 

A110V mutant has also been reported to cause resistance to INH. The larger side chain of valine alters the H108 conformation, leading to an inefficiency in the binding of INH while also maintaining its enzymatic activity (Wei et al., 2003). Other mutations such as substitution T275P lead to protein instability, which produces an unfolded protein, which is neither capable of activating INH nor catalyzing its reaction (Saint-Joanis et al., 1999). 

Since 2017, several new single nucleotide polymorphisms (SNP) for KatG which are associated with INH resistance have been isolated, and some are summarized herein (Table S1) (Figure 4A). Thwe et al. (2021) performed a study using PCR and DNA sequencing in 65 drug-resistant Mtb isolates, revealed a number of new mutations on *katG* (Thwe *et al*., 2021). A novel single nucleotide polymorphism (SNP) that causes INH resistance was identified in two of the studied isolates. This mutation is the substitution of proline for arginine at position 365 (P365R) (Thwe *et al*., 2021). This mutation was not characterized, however, although the residue P365 is more than 16Å away from the KatG active site, it was assumed to cause INH resistance. The substitution of proline for arginine probably causes conformational changes that lead to a displacement of key secondary structure elements leading to a lower binding affinity for INH. In this study, only KatG has been evaluated and other mutations in different genes could also be present difficulting the categorization of this mutation as the unique mechanism of resistance to INH.

Kandler et al. (2018), also analyzed 52 INH^R^ Mtb strains using whole-genome sequencing (WGS) and identified 5 novel mutations in KatG, including those that lead to residues substitutions W121Q, W161R, E402stop, A480del, L415P (Kandler *et al*., 2018). Islam et al. (2019) performed a large-scale susceptibility test using 10 drugs, including INH, RIF and EMB, against 206 clinical isolates. For KatG, several novel mutations involved in INH resistance were identified, particularly C20R, G33V, W91stop, W91R, P92S, G111S, G125S, Q127P, D142G, L147P, S211G, G279V, A312P, H417Q, V431A, L436P, Q461P, G466R, G490S, V581G, N508D, E607A and N660D, (Islam et al.2019). Interestingly, the mutations G111S (7.9 Å), Q127P (12.5 Å), D142G (10.7 Å), G279V (10.3 Å) and A312P (8.0 Å) are the closest to the heme group binding cavity (Islam *et al*., 2019) and should have an impact in INH activation. These mutations have not been validated through genetic and functional experiments, but may represent great prospects for INH resistance markers.

WGS was also used for the drug resistance prediction in 137 drug-resistant Mtb isolates from Shanghai and 78 from Russia. This study also identified a novel KatG mutation, S17G, which has relevance to INH resistance (Wang et al., 2022). The N-terminal region of KatG seems to be involved in inter-domain interactions, which is important to dimerization (Bertrand *et al*., 2004). The mutations S17G, C20R and G33V may cause instability of the protein dimerization. Residues 278 to 312 have been reported to be part of a loop that is possibly involved in the INH binding site (Bertrand *et al*., 2004) and then, the mutation G279V should also be involved in decreasing the INH binding affinity but not altering the KatG function. More studies are necessary to categorize those novel mutations as resistant conferring and to determine the resistance mechanism, although the WGS strategy has had success in predicting resistant-conferring mutations in Mtb (Kandler et al., 2018).

Alongside *katG* mutations, other mutations often found and reported to confer INH resistance are those in the *inhA* promoter region, such as T(− 8)G/A, C(− 15)T and A(− 16)G. These mutations cause overexpression of InhA rather than structural protein modifications (Musser et al., 1996; Ramaswamy and Musser, 1998; Vilchèze et al., 2006) (Figure 4C). According to a data compilation of *inhA* mutations performed by Seifert et al. (2015), in which they analyzed more than 11000 isolates, mutations in *inhA* promoter region, particularly -15 and -8 were observed in approximately 20,5% of the 6,192 phenotypically resistant isolates, while amino acid substitutions on *inhA* coding region were found in only approximately 1% of the analyzed strains (Seifert *et al*., 2015). Those mutations involved in the coding region and that are clinically relevant include K8N, I16T, I21T, I25T, I47T, A78V, S94A, S94R, I95P, L168W, A190S, I194T, R202G, E217D, T241M, D256N, I258T, I258V and Y259H (Figure 4C) (Vilchèze *et al*., 2006; Vilchèze and Jacobs, 2014; Seifert *et al*., 2015). Missense mutations affecting *inhA* usually cause an influx of water molecules into the INH-NAD binding site, which decreases NAD-INH adduct and NADH binding affinity. Due to the lower binding affinity, the enzyme turnover rate is altered, changing the bound time of the coenzyme and adduct in the InhA active site. This effect increases the proportion between unbound InhA and bound InhA, and INH-NAD and NADH renovation rate will be higher for the mutants compared to the WT InhA. As consequence, the higher turnover guarantees certain activity of the enzyme even in the presence of INH-NAD (Dessen et al., 1995; Oliveira et al., 2006; Vilchèze *et al*., 2006; Dias et al., 2007). Several mutations, including I21T, I47T, S94A and I95P were biochemically or biophysically characterized. For most of these mutant proteins, it has been observed that the K_d_ (dissociation constant) for NADH is much higher than for the wild-type enzyme. Interestingly, the mutant I95P did not show any activity in vitro, which indicates that the protein-protein complex formation of the enzymes from the FAS-II system could be important for its residual activity (Basso et al., 1998). The substitution S94A is the most structurally and functionally characterized. Ser94 hydroxyl side chain performs an indirect hydrogen bond with NADH through a water molecule. In the mutated protein, this water molecule is lacking because of the apolar side chain of alanine. As a consequence, there is a direct impact on the affinity of the coenzyme to InhA (Dessen *et al*., 1995; Oliveira *et al*., 2006; Vilchèze *et al*., 2006; Dias *et al*., 2007). On the other hand, Oliveira *et al*., 2006 and Dias *et al*., 2007 further biochemically or structurally investigated the mutations I21V and I47T and observed an altered NADH dissociation constant and a perturbation in water molecules in the active site of the mutant enzymes in comparison to the wild-type enzyme (Oliveira *et al*., 2006; Dias *et al*., 2007). Although the performed biophysical studies in a few InhA mutants have shown similar molecular resistance mechanisms, others should exist since there are mutations covering the whole extension of the InhA primary structure (Figure 4C) (Vilchèze *et al*., 2006; Vilchèze and Jacobs, 2014; Seifert *et al*., 2015). In addition, novel mutations in the *inhA* gene have continually appeared, such as the recent identification of substitution G141E, which has been identified in a resistant Mtb isolate harboring KatG mutation D142G and EthA S266R, but has not been validated yet as resistant-conferring through genetic and functional assays. (Islam et al., 2019).

## Resistance to RIF

Rifampicin (RIF), also known as Rifampin, is an antimicrobial chemically derived from the rifamycins, which are members of the ansamycins antibiotic family. Rifamycin compounds consist of 7 different molecules, in which RIF is a derivative of Rifamycin SV. Rifamycin SV chemical structure was altered by the synthetical addition of a 3-(4-methyl-1-piperazinyl)-iminomethyl group, improving chemical stability and oral administration while maintaining high antibacterial activity (Murray et al., 2015; Brucoli and McHugh, 2021; Zloh et al., 2021).

RIF was first synthesized in 1965 and since 1970 has been used for TB treatment, particularly in combined therapy with INH (Grobbelaar et al., 2019). The addition of RIF to the treatment regimen together with INH and EMB also enabled the therapy to be shortened from 12 to 9 months because of its higher efficacy in sterilization. Thus, currently, RIF is considered a first-line drug against TB, and part of the standard drug combination used for TB treatment (Murray et al., 2015; Grobbelaar *et al*., 2019; World Health Organization, 2021).

RIF inhibits RNA synthesis by binding to the DNA-dependent -RNA polymerase (RNAP) (McClure and Cech, 1978). RIF binds to the β-subunit of RNAP, encoded by the *rpoB* gene, and causes a steric clash between the 5’ phosphates elongating RNA. Thus, RIF inhibits the RNA elongation path for RNA transcripts of 2 or 3 nucleotides in length during the translocation movement of RNAP. This blockage severely disturbs the bacterial transcription mechanism and consequently leads to cell death (McClure and Cech, 1978; Campbell et al., 2001; Zloh et al., 2021).

**Figure 5 -:**
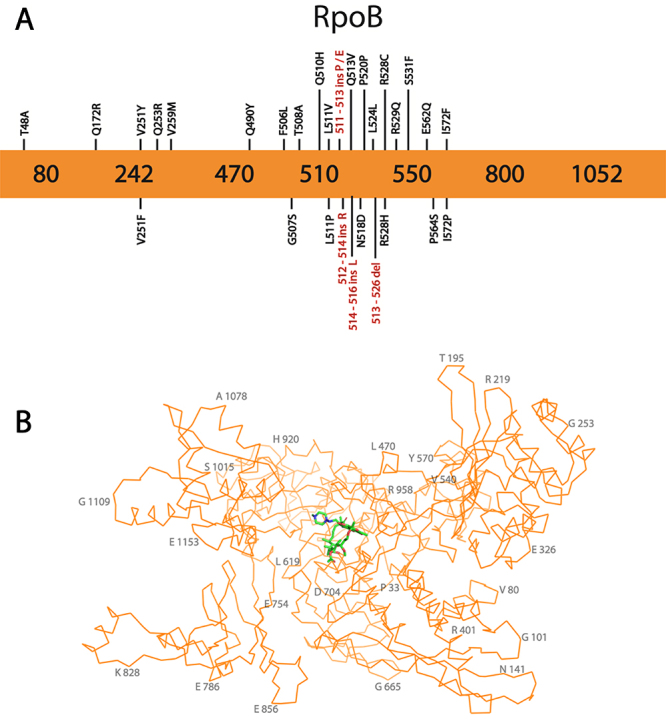
Structure and key mutations for β-subunit of RNAP encoded by *rpoB* gene, the main target of RIF and the most reported protein in RIF resistance. **A -** Representation of β-subunit of RNAP primary sequence in orange, showing the most relevant mutations. Substitutions are shown in black, while insertions and deletions are shown in red. **B -** The β-subunit of RNAP structure. The representation in orange shows the Cα contours while RIF is shown with carbon atoms in light green. The β-subunit of RNAPR encoded by *rpoB* was obtained from PDB entry 5UHD (Lin et al., 2017) and the figure was prepared using the software PyMOL (Schrödinger, 2015). Some residues are numbered in 50% opacity for better visualization.

RIF resistance is mainly associated with mutations in the *rpoB* gene, which encodes the subunit β of RNAP (Figure 5). RNAP is an essential protein, and its sequence is extremely conserved in all bacteria (Campbell et al., 2001; Betts et al., 2002; Goldstein, 2014). It has been extensively reported that the region between codons 426 to 452 in Mtb and 507 to 533 in *E.coli* of the *rpoB* gene is more commonly affected by mutations and these are often involved in RIF resistance. This region contains 81 bp and is usually referred to as R-resistance determining region (RRDR). The amino acid chain encoded by RRDR was confirmed to be important to RIF binding and consequently, mutations in this region affect the affinity of RIF to RNAP (Campbell *et al*., 2001; Pang et al., 2013; Goldstein, 2014). Thus, about 95% of all RIF-resistant (RIF^R^) strains have mutations in this region, particularly at codons 516, 526, and 531 (435, 445, and 450 in Mtb), which are responsible for nearly 90% of all known RIF^R^ strains (Figure 5) (Ohno et al., 1996; Somoskovi et al., 2001; Campbell *et al*., 2001; Goldstein, 2014). 

This mechanism of resistance can be exemplified by the substitutions S531L, which is the most common substitution or by H526D (Figure 5). Both of these mutations interfere with intramolecular hydrogen bonds. The side chain of the mutant L531 disrupts the RIF binding because of a structural clash with RIF binding position, according to Pang et al., 2013. On the other hand, D526 causes a charge repulsion decreasing the RIF binding affinity (Mcnerney et al., 2000; Mikhailovich et al., 2001; Pang *et al*., 2013). Other substitutions that are also involved in RIF resistance, but usually have a higher phenotypic variation, including those showing a lower level of resistance, are H526L, H526G, H526R and L533P (Mcnerney *et al*., 2000; Mikhailovich *et al*., 2001; Pang *et al*., 2013). In addition, Hirani et al. 2020 also identified from an MDR-TB isolate from India, a *rpoB* mutation that encodes an insertion of an arginine between the residues 512 and 513 (512-Arg-514) (Hirani *et al*., 2020). Interestingly, other insertions in the RRDR region are also commonly reported and associated with RIF resistance, particularly 514 L 516, 511 P 513, 511 E 513 (Sinha et al., 2020) (Figure 5A). 

A number of substitutions have also been reported to change other residues outside the RRDR. In the last 5 years, RIF^R^ strains have been continually analyzed, and many new *rpoB* mutations were identified (Table S2). Takawira et al. (2017) collected 100 isolates from Zimbabwe, and detected 13 novel mutations, varying from substitution mutations inside RRDR, such as R529Q, and also outside of RRDR, such as I572P that have not yet been validated as resistant-conferring mutations but have been identified in resistant strains. (Takawira *et al*., 2017) (Figure 5). Maningi et al. (2018) also conducted a study using 240 isolates from South Africa and identified 5 mutations outside of the RRDR, of which 2 of them are associated with RIF resistance, T480A, and Q253R (Maningi *et al*., 2018). Finally, more recently, a WGS was used for the prediction of drug resistance in 137 drug-resistant Mtb isolates from Shanghai and 78 from Russia. This study has identified a novel *rpoB* mutation that encodes rpoB with a substitution Q172R (Wang et al., 2022). Although more studies are necessary to categorize this novel mutation as resistant conferring and to determine the resistance mechanism, the WGS strategy has had success in predicting resistant-conferring mutations in Mtb (Kandler et al., 2018).

Additionally, approximately 5% of all (RIF^R^) strains do not have mutations in *rpoB* gene at all. Several studies propose that the mechanism of RIF could be the overexpression of genes involved in bacterial efflux mechanisms, such as *mmr, mmpL7, Rv1258c, p55* and *efpA*. This hypothesis indicates that resistance in Mtb can emerge from a combination of specific mutations and bacterial metabolic adaptations (Machado et al., 2017).

In the context of drug susceptibility tests (DST) for Mtb, it is important to highlight that although phenotypic DST methods are the “gold standard” for the detection of Rifampicin resistance-conferring mutations, there are some mutations that are consistently missed by those methods, which is the case for mutations conferring low-level RIF resistance, commonly referred as disputed mutations. These mutations, which include L511P and D516Y, are associated with poor clinical outcomes but evade the detection of phenotypic DST screenings. In such circumstances, genotypic DST, such as WGS, is promising for the detection of disputed mutations. Genotypic DST can also provide important information regarding compensatory mutations that restore bacterial fitness due to a decrease in this characteristic caused by mutations in the RRDR of *rpoB*. These compensatory mutations were reported outside of RRDR in *rpoB*, or even in other genes, such as *rpoA/C*, and can directly impact the clinical outcome for such strains (Ahmad and Mokaddas, 2014; Miotto et al., 2018; Al-Mutairi et al., 2019a,b; Ma et al., 2021; Shea et al., 2021). 

## Resistance to PZA

Pyrazinamide (PZA) is an analog of nicotinamide, with the substitution of the pyridine group for pyrazine. This drug was first synthesized in 1936, but it was only acknowledged as a potential drug for the treatment of TB in 1952. Since 1970, PZA has been used as a first-line drug for TB treatment. PZA is the unique anti-TB drug that is selective against latent TB and the addition of this drug to the previous TB treatment regimen, composed of INH, RIF and EMB, was essential for the TB treatment regimen reduction from 9 to 6 months (Fox et al., 1999; Murray et al., 2015).

Pyrazinamide (PZA) is an analog of nicotinamide, with the substitution of the pyridine group for pyrazine. This drug was first synthesized in 1936, but it was only acknowledged as a potential drug for the treatment of TB in 1952. Since 1970, PZA has been used as a first-line drug for TB treatment. PZA is the unique anti-TB drug that is selective against latent TB and the addition of this drug to the previous TB treatment regimen, composed of INH, RIF and EMB, was essential for the TB treatment regimen reduction from 9 to 6 months (Fox et al., 1999; Murray et al., 2015).

**Figure 6 -:**
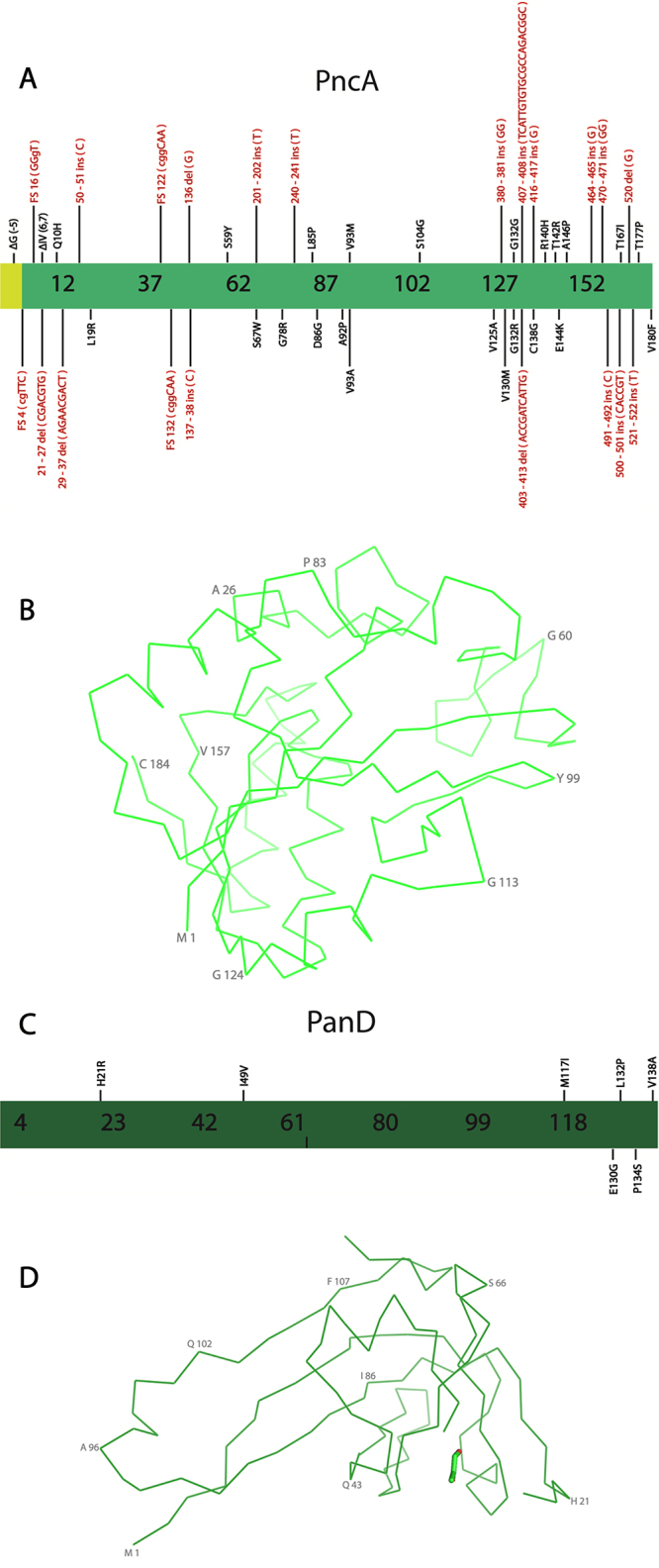
Structure and relevant mutations for PncA and PanD, enzymes involved in PZA resistance. **A -** Representation of the PncA primary sequence in light green, showing key mutations. Substitutions are shown in black, while insertions and deletions are sown in red. The *pncA* promoter region is shown in yellow. **B -** Structure of PncA. The contours of Cα are shown in light green. PncA structure was obtained from PDB entry 3PL1 (Petrella et al., 2011) and the figure was prepared using the software PyMOL (Schrödinger, 2015). Some residues are numbered in 50% opacity for better visualization. **C -** Representation of the PanD primary sequence is shown in dark green, highlighting the most significant mutations. **D -** PanD Structure. The contours of Cα are shown in dark green, while PZA is shown with carbon atoms in light green. The PanD Structure was obtained from PDB entry 6OZ8 (Sun et al., 2020) and the figure was prepared using the software PyMOL (Schrödinger, 2015)**.** A number of residues are numbered in 50% opacity for better visualization.

PZA, similarly to INH, is also a prodrug and needs to be converted to pyrazinoic acid (POA) by the nicotinamidase or pyrazinamidase (PZAse) (Figure 6), encoded by the *pncA* gene, to exhibit its antitubercular activity (Scorpio and Zhang, 1996). PZA/POA shows lesser activity in growing bacteria, and greater, in the persistent or latent stage, having an important role as a sterilizing drug (Mccune and Tompsett, 1956; Mitchison, 1985; Hu et al., 2006). 

The most accepted mechanism of action for this drug is that in acidic environments, such as inflamed tissues as a consequence of Mtb activity, the POA influx into the bacterial cell is facilitated, leading to POA accumulation inside the bacillus and the eventual cytoplasm acidification (Zhang et al., 2014). POA also seems capable of disrupting membrane potential and of de-energizing the membrane (Zhang *et al*., 1999, 2003). POA may also be involved in the inhibition of the trans-translation process through the binding to protein RpsA (Shi et al., 2011). 

In recent years, strong evidence indicated that the enzyme PanD is one of the major targets for PZA/POA (Sun et al., 2020). PanD (Figure 6) is an enzyme involved in the CoA and pantothenate biosynthesis, being directly involved in β-alanine production (Zhang et al., 2003, 2014). CoA and pantothenate are key molecules to Mtb persistence, which may explain why PZA/POA is more active for bacteria in these conditions (Zhang *et al*., 2002; Sambandamurthy et al., 2002). POA binds to the PanD active site and competes with its substrate, D-aspartate. The Mtb PanD crystal structure was solved in complex with POA, which revealed the interactions of this ligand and the protein. Particularly, it was revealed that POA performs key hydrogen interactions with A74, A75, R54 from the PanD active site. (Sun *et al*., 2020).

Mutations that cause resistance to PZA in Mtb have been observed predominantly in *pncA* gene impacting the PZase activity, leading to a decrease in the efficiency of PZA activation (Konno et al., 1967; Petrella et al., 2011) (Figure 6B); however, a number of mutations have also been observed in *panD* and *rpsA* genes (Shi et al., 2011; Feuerriegel et al., 2013; Sun et al., 2020). In the case of mutations on PanD, PZA resistance should be caused by a lower POA affinity and decreasing the residence time on PanD active site. Particularly, mutations on PanD residues of two loops that cover the active site have been reported. These PanD loops are formed by residues 20-24 and 119-126, which are involved in the formation of a barrier over the protein active site to maintain the substrate isolated from the solvent (Figure 6D) (Sun *et al*., 2020). 

Sun et al. (2020) confirmed that the PanD mutations H21R and M117I are associated with PZA resistance. These researchers investigated using isothermal titration calorimetry (ITC) and enzymatic assays the affinity and the activity of these two mutants and they observed higher affinity of POA and stronger inhibition for the wild-type enzyme in contrast to the mutants (Sun *et al*., 2020). Using crystallography, it was also confirmed that the mutations H21R and M117I affect regions that are near the C-terminal loops of the α and β-chains. Still, Hameed et al. (2020) reported the identification of a novel PanD mutation, L132P, isolated in clinical isolates from Southern China. The mutation L132P is interesting due to its proximity to the PanD C-terminal loop (Figure 6D). However, currently, there are no biochemical or biophysical characterization studies for this mutation (Hameed *et al*., 2020). Other clinically important mutations for PanD are H21R, I49V, E130G, P134S, and V138A H21R, I49V, E130G, P134S, and V138A (Zhang et al., 2013), but there are also no functional studies about the effect of these substitutions on the enzyme and ligand affinities (Figure 6D).

Although mutations can also occur through whole *pncA* extension, this gene has three major regions that are mostly affected by mutations and polymorphisms which are: Nucleotides 3-17, 61-85 and 132-142 (Scorpio et al., 1997; Zhang et al., 2014). These regions seem to be part of three different loops that are key regions of the active site architecture. Daum et al. (2019) performed a study using 91 Mtb clinical isolates from Ukraine, and identified a number of mutations in *pncA* gene. 11 novel mutations were identified and those can be divided by substitution mutations: Q10H, V93M, G132R, A146P, T177P; deletion mutations: Promoter Δ(-5), ΔIV(6,7)(deletion of isoleucine and valine at positions six and seven, respectively); frameshift insertions: 4 frameshift (cgTTG), 16 frameshift (GGgT), 132 frameshift (cGGT); and insertions: 122 frameshift (cggCAA). Of these mutations, ΔIV(6,7), Q10H, and V93M were confirmed to cause resistance to PZA (Figure 6B) (Daum *et al*., 2019). 

Since 2017, several new mutations for PncA that cause PZA resistance have been isolated, and some are summarized herein (Table S3). Khan et al. (2018) biophysically characterized three PncA mutations, including L19K, R140H and E144K. Using molecular dynamics simulations, these authors observed that the RMSD, obtained through superposition, for the mutant protein models seems to be higher than 2Å when compared to the wild-type (WT) position. This suggests that these mutations disturb the protein stability and reduce its activity against PZA strongly contributing to resistance (Khan *et al*., 2018). For these mutants, the hydrogen bonding of the PZAse with POA was disturbed in comparison to the wild-type protein, indicating changes in residues that are part of the ligand-binding site (Khan *et al*., 2018). 

Li et al. (2021) performed a study using 465 clinical isolates, 424 of those being drug-resistant, identified 30 novel mutations involved with PZA resistance (Table S3). Among them, 24 were confirmed to not have Pzase activity, while 6 maintained it. Those first 24 mutations occur in *pncA* regions involved in PZase activity, while the other 6 mutants have amino acid substitution in other regions. 15 negative Pzase active mutations were tested for PZA susceptibility, and all were classified as resistant, while the two Pzase positive active mutations were susceptible to PZA (Li *et al*., 2021).

## Resistance to EMB

Ethambutol (EMB) was identified as a drug with potential anti-TB activity in 1960, and it has synergy with INH and it is part of the drug combination used in TB standard treatment, which also includes PZA and RIF. EMB has a relatively simple chemical structure, consisting of an ethylenediamine molecule at the center, with the addition of butanol moieties at the end of each side of the carbon chain (Thomas et al., 1961; Murray et al., 2015).

**Figure 7 -:**
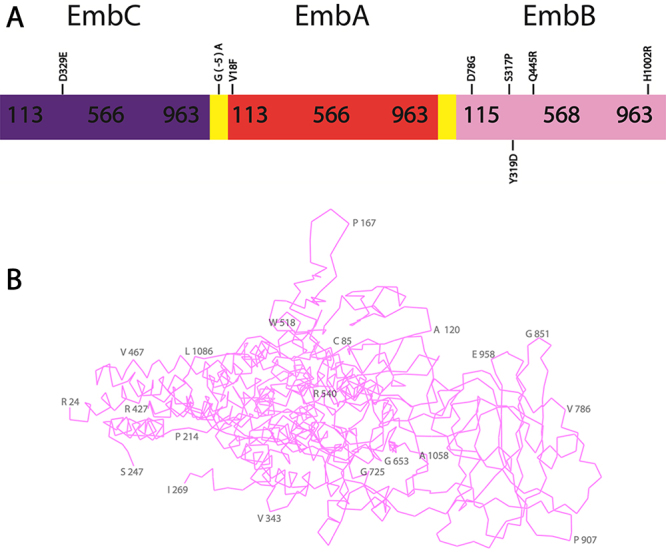
Structure and key mutations involved in the *embCAB* operon and their proteins, which are involved with EMB resistance. **A -** Representation of *embCAB* operon and the position of mutations involved in the resistance to EMB. The EmbC, EmbA and EmbB sequences are represented in purple, red and pink, respectively, showing the mutations described herein. The intergenic spaces between the gene encoding regions are shown in yellow. **B -** EmbB Structure. The Cα contours are shown in pink. EmbB structure was obtained from PDB entry 7BVF (Zhang et al., 2020) and the figure was prepared using the software PyMOL (Schrödinger, 2015). A number of residues are numbered in 50% opacity for better visualization.

The EMB mechanism of action involves the inhibition of arabinose incorporation at the arabinogalactan from the mycobacterial cell wall, leading to the cell accumulation of decaprenyl-phosphate-arabinose that eventually culminates in bacterium cell death (Takayama and Kilburn, 1989; Sreevatsan et al., 1997; Telenti et al., 1997). The operon *embCAB* encodes the proteins EmbA, EmbB and EmbC, which are arabinosyltransferases involved in the cell wall arabinan biosynthesis (Belanger et al., 1996; Telenti *et al*., 1997) (Figure 7). EmbA and EmbB are associated to produce a heterodimeric complex, while EmbC is a homodimeric enzyme (Tan et al., 2020; Zhang et al., 2020; Bendre et al., 2021). Figure 7B shows the structure of EmbB, which has the most key mutations involved in EMB resistance. The cryo-EM tridimensional structures of these three proteins in complex with their glycosyl donor substrate, decaprenyl-phosphate-arabinose; the acceptor substrate, diarabinose; and the known inhibitor, EMB, have been determined, and it was confirmed that EMB binds competitively to the active sites of EmbB and EmbC (Goude et al., 2009; Tan *et al*., 2020). An acyl carrier protein, AcpM, was also attached to the Emb proteins. The complex EmbA-EmbB-AcpM catalyzes the transfer of an arabinose molecule from the acceptor to the donor producing a 1-3 linkage between the arabinose residue and the arabinan receptor, while the complex EmbC-Acp)M is involved in the extension of the arabinan chain, following a 1-5 linkage (Zhang *et al*., 2020; Bendre *et al*., 2021). EMB was shown to inhibit these reactions occupying the disaccharide product binding site (Wolucka et al., 1994).

Additionally, EMB has synergistic activity with INH. It is proposed that EtbR, a transcription factor encoded by the *Rv0273c* gene, is responsible for negatively regulating InhA expression, acting as a repressor for InhA expression. It was proposed that EMB is capable of interacting with this repressor, EtbR, amplifying its effect. This ultimately would lead to even less InhA expression, and consequently, higher INH susceptibility, in the co-administration with EMB (Zhu et al., 2018).

The EMB resistance mostly involves mutations in the *embCAB* operon*,* which encodes target proteins of EMB. Even so, in 30% of EMB-resistant (EMB^R^) strains, mutations in *embB* gene are not reported (Zhang and Yew, 2015). Mutations in *embC* and the *embC-embA* intergenic space were also reported to EMB resistance (Cui et al., 2014). 

Mutations on the *embC* gene may lead to the production of mutant proteins with a lower EMB binding affinity due to substitutions near the protein binding site to EMB, while mutations in *embC-embA* intergenic space may have a strong impact on the mRNA expression of *embA* and *embB* genes (Cui et al., 2014; Zhang et al., 2020). 

Mutations in the residue M306 from EmbB have been identified in more than 68% of EMB^R^ isolates (Safi et al., 2008; Zhang and Yew, 2015). Furthermore, G406 and Q497 have also been reported as resistance hotspots and mutations in these sites are frequently isolated in EMB^R^ strains (Plinke et al., 2010; Dai et al., 2019). Based on the structure of EmbB, M306 is directly involved in EMB binding, and mutations affecting this residue, or those residues that interact with it, including Y302 and E327, disturb the EMB binding affinity because of differences in the interaction between the protein active site and EMB (Plinke *et al*., 2009, 2010; Zhang *et al*., 2020) (Figure 7). On the other hand, mutations affecting Q497 may interfere with the interaction of E327 and EMB, which decreases the binding affinity of this drug. Finally, the resistance mechanisms involved in mutations affecting G406 are proposed to lead to a steric hindrance and consequently cause conformational changes in EMB binding site, decreasing the ligand affinity (Plinke *et al*., 2009, 2010; Zhang *et al*., 2020).

Since 2017, a number of mutations in proteins encoded by the *embCAB* operon have been reported (Table S4). Li et al. (2017) identified 54 resistant Mtb isolates after performing susceptibility tests from Yunnan, China. These authors identified a novel mutation for *embB*, which encodes the substitution D78G (Li *et al*., 2017) (Figure 7A). However, a closer analysis of the position of this residue indicates that it is not involved in the protein active site and it has not been validated through gene replacement phenotypic assays as a resistance-conferring mutation. 

Park et al. (2018a) analyzed 34 resistant isolates from Daejeon City in South Korea and reported two novel EmbB mutations, S317P and Q445R (Park *et al*., 2018a). Still, Park *et al*. (2018b) also identified two novel *embB* mutations from 30 MDR-TB isolates from Cheongju, Korea, that encodes the substitutions of Y319D and H1002R (Park *et al*., 2018b). Those mutations have been correlated with EMB resistance since phenotypical DST of the isolates showed resistance, but were not yet validated through gene replacement (Figure 7A).

Sun et al. (2018) tested 125 isolates from China and measured the MIC of these isolates against EMB. The authors identified 2 novel mutations for gene *embA:* one which is located in the intergenic region, *embA*G(−5)A, and the other one that encodes the substitution V18F (Figure 7A). These authors also identified a novel mutation in *embC* that encodes the substitution D329E (Sun *et al*., 2018). However, the mutations *embA*G(−5)A and V18F were identified in isolates that also have other e*mbB* mutations, which are well known to be involved in EMB resistance (Figure 7A). Although mutations in the intergenic region of *embA-embC* are known to upregulate the expression of proteins encoded by *embCAB* operon, there is no validation whether these related mutations are solely involved in EMB resistance (Cui et al., 2014; Sun *et al*., 2018). 

In the context of DST for EMB, it has been reported that phenotypic DST methods have a higher percentage of false susceptibility results for EMB^r^ strains than genotypical and molecular DST methods. The lower sensitivity of the phenotypical DSTs is mainly explained due to the slower activity of EMB for Mtb, and the even lower activity of the drug in the culture media used, which explains EMB^r^ strains harboring mutations in codons 306 or 406 categorized as susceptible, while it is highly unlikely that those mutations are not associated with EMB resistance (Ahmad et al., 2007; Ahmad *et al*., 2016; Al-Mutairi et al., 2019b). 

## Resistance to other drugs

Bedaquiline (BDQ) was one of the last approved drugs by the FDA to combat TB after a void of about 30 years. This drug has antimycobacterial activity based on the inhibition of mycobacteria ATP synthase without affecting the human enzyme. BDQ is a diarylquinolone, structurally organized with amine and alcohol side chains (Patel et al., 2019; Khoshnood et al., 2021). Canonically, ATP synthase is formed by a membrane domain, F0, with the subunits a, b2 and c10, and by a cytoplasmatic domain, F1, which is constituted by the subunits a3, b3, g, d and ε (Khoshnood *et al*., 2021). BDQ inhibits ATP synthase by the binding in the F0 domain of the Mtb ATP synthase, particularly in the subunit c and subunits a-c interface, specifically interacting with the residues L63, E65, A66, A67 Y68 and I70 from c subunit and L70, P172 and I173 from the a subunit (Guo et al., 2021).

BDQ resistance has already been observed and it is commonly associated with mutations in specific genes, including *atpE*, which encodes the subunit c of ATP synthase and the transcription regulation factor gene *mmpR* (Li et al., 2019; Kadura et al., 2020; Battaglia et al., 2020; Goossens et al., 2021). These mutations include D28V, E61D, A63P and I66M in ATP synthase subunit c, and mutations W42R, and S53L for MmpR. It has also been observed frameshifts in the region between the codons 38-144 for the *mmpR* gene (Kadura *et al*., 2020). Nevertheless, Ismail *et al*. (2021) summarized 14 *atpE* and 237 *mmpR* polymorphisms reported in the literature between 2006 to 2020. From these, many mutations were associated with BDQ resistance, including *atpE* mutations that were reported in clinical BDQ-resistant strains. However, further confirmations of such polymorphisms are those solely responsible for the BDQ resistance still need to be confirmed. (Ismail *et al*., 2021). 

Pretomanid (Pa) and Delamanid (DLM) are nitroimidazole antimycobacterial agents, with a nitroimidazooxazine core. Pa is currently used in association with BDQ and Linezolid (LZD) against MDR-TB. Pa and DLM mechanism of action involves the inhibition of mycobacterial mycolic acids and cell wall biosynthesis. Nitroimidazoles are usually activated by the enzyme Ddn, which binds the unusual co-factor F_420_. Pa and DLM showed antimycobacterial activity against replicating and non-replicating bacteria (Li *et al*., 2019). One of the proposed mechanisms of action for Pa and DLM in replicating bacteria is the oxidation of precursors necessary for lipid biosynthesis, inhibiting this process. For non-replicating bacteria, followed by the Ddn activation of Pa/DLM, these drugs might generate reactive nitrogen species, such as nitric oxide that can interact with cellular components and lead to bacterial death (Keam, 2019; Stancil et al., 2021; Hori et al., 2022). 

In mutant Mtb strains, Pa and DLM capacity for generating reactive substances is inefficient (Guglielmetti et al., 2020; Stancil et al., 2021). Therefore, the more commonly reported resistance mechanism associated with Pa and DLM involves mutations to key enzymes responsible for nitroimidazole activation, including Ddn and the further enzymes Fgd1, FbiA, FbiB, FbiC and FbiD (Parveen et al., 2021). Several groups have already described a number of mutations in the genes that encode these proteins, including Ddn S11stop and Y133D; fbiC V720I and P372S and fbiD A198P for Pa and Ddn D241A, G242A, W88stop and L107P for DLM (Pang et al., 2017; Kidwai et al., 2017; Yang et al., 2018; Kadura et al., 2020; Rifat et al., 2021).

Linezolid (LZD) is an oxazolidinone drug with antimicrobial activity by the inhibition of protein synthesis, particularly binding to rRNA 23S from ribosomal subunit 50S. LZD inhibits the formation of the ribosomal complex 70S, leading to a non-functional initiation complex, and, consequently, decreasing the efficiency of the translation process (Hashemian et al., 2018; Fermeli et al., 2020). Structurally, LZD is formed by an N-aryl ring core, which is important to its function as an antimicrobial. LZD was clinically introduced in 1996, and in the last 10 years, its activity against Mtb has been evaluated. Usually, this drug has been repurposed for anti- TB treatment, particularly in cases of MDR-TB and XDR-TB. LZD also seems to possess anti- TB activity against persistent bacteria (Drusano et al., 2018). 

The resistance mechanism for LNZ involves, primarily, mutations to ribosomal protein L3 and subunit 23S, encoded by genes *rplC* and *rrl*, respectively. The mutation C154R in RplC is one of the most prevalent ones (Ushtanit et al., 2021). Nevertheless, other mutations reported to LZD were summarized by Kadura et al. (2020) and Khosravi et al. (2021). The other prevalent mutations include G2270T, G2299, G2746A, G2814T and C2848A for Rrl. These authors also identified 2 polymorphisms in LZD resistance strains for rplC, V141I and I150N. However, they have not been completely validated to be the unique reason for LZD resistance (Kadura *et al*., 2020; Khosravi *et al*., 2021).

Clofazimine (CFZ) is a drug repurposed for resistant TB treatment. Structurally, this molecule is a riminophenazine, constituted of a phenazine core with an *R-*imino group. The mechanism of action of this molecule is not entirely understood, as the envelope, the respiratory chain, or even the DNA were reported as possible targets (Mirnejad et al., 2018; Lange et al., 2019). 

The most important mechanism of resistance associated with CFZ includes mutations in the gene *mmpR* which encodes a transcript regulator of efflux pumps. The most prevalent substitutions include R156*, G193del, and G193Ins (Kadura et al., 2020).

## Conclusions

In summary, this review compiles the molecular mechanisms of resistance and also describes a number of novel mutations identified in the last five years for the first-line drugs (INH, PZA, RIF and EMB) that are currently used for TB treatment regimens. Identifying, reporting, and understanding the resistance of Mtb to these antimicrobials has pivotal importance because of the difficulty in the management and treatment of TB, particularly those caused by MDR and XDR strains. With the rise of novel methods of genome sequencing and the development of structural biology, particularly cryo-EM, the identification and understanding of the mechanism of resistance has considerably evolved in the last five years allowing the anticipation of the mutational effect. Overall, all advances made in this field might considerably contribute to more personalized use of anti-TB drugs, avoid the dissemination of resistant strains, better management of the antimicrobials used in TB treatment, and the discovery of new medicines. 

## Supplementary material

The following online material is available for this article:

Table S1 - Novel KatG mutations.

Table S2 - Novel RpoB mutations.

Table S3 - Novel PncA mutations.

Table S4 - Novel EmbC, EmbA, EmbB mutations.
