# Supplementary material for: Mutations and insights into the molecular mechanisms of resistance of Mycobacterium tuberculosis to first-line
Source: Genet Mol Biol. 2023 Jan 23;46(1 Suppl 2):e20220261. doi: 10.1590/1678-4685-GMB-2022-0261 (PMC9887390; doi:10.1590/1678-4685-GMB-2022-0261)
Supplement: Table S1 - [file 1415-4757-GMB-46-1-s2-e20220261-s1.pdf]

Genetics and Molecular Biology
DOI: https://doi.org/10.1590/1678-4685-GMB-2022-0261

Supplementary Material to "Mutations and insights into the molecular mechanisms of resistance of Mycobacterium tuberculosis to first-line drugs"

Table S1 - Novel KatG mutations.

Mutation	Reference
P365A	(Thwe *et al*., 2021)
W121Q<br>W161R<br>E402stop<br>A480del<br>L415P	(Kandler *et al*., 2018)
KatG S17G	(Wang *et al*., 2022)
A312P<br>N660D<br>L147P<br>C20R<br>D142G<br>S211G<br>W91R<br>V581G<br>G466R<br>G279V<br>L436P<br>N508D<br>P92S<br>G125S<br>Q127P<br>V431A<br>G490S<br>Q461P<br>E607A<br>H417Q<br>G111S<br>G33V<br>W91stop	(Islam *et al*., 2019)

References

Islam MM, Tan Y, Hameed HMA, Liu Z, Chhotaray C, Liu Y, Lu Z, Cai X, Tang Y, Gao Y *et al.* (2019) Detection of novel mutations associated with independent resistance and cross-resistance to isoniazid and prothionamide in *Mycobacterium tuberculosis* clinical isolates. Clin Microbiol Infect 25:1041.e1-1041.e7.

Kandler JL, Mercante AD, Dalton TL, Ezewudo MN, Cowan LS, Burns SP, Metchock B, Cegielski P and Posey JE (2018) Validation of novel *Mycobacterium tuberculosis* isoniazid resistance mutations not detectable by common molecular tests. Antimicrob Agents Chemother 62:e00974-18.

Thwe EP, Namwat W, Pinlaor P, Rueangsak K and Sangka A (2021) Novel mutations detected from drug resistant *Mycobacterium tuberculosis* isolated from North East of Thailand. World J Microbiol Biotechnol 37:194.

Wang L, Yang J, Chen L, Wang W, Yu F and Xiong H (2022) Whole-genome sequencing of *Mycobacterium tuberculosis* for prediction of drug resistance. Epidemiol Infect 150:e22.
