# Supplementary material for: Mutations and insights into the molecular mechanisms of resistance of Mycobacterium tuberculosis to first-line
Source: Genet Mol Biol. 2023 Jan 23;46(1 Suppl 2):e20220261. doi: 10.1590/1678-4685-GMB-2022-0261 (PMC9887390; doi:10.1590/1678-4685-GMB-2022-0261)
Supplement: Table S2 - [file 1415-4757-GMB-46-1-s2-e20220261-s2.pdf]

Genetics and Molecular Biology
DOI: https://doi.org/10.1590/1678-4685-GMB-2022-0261

Supplementary Material to "Mutations and insights into the molecular mechanisms of resistance of Mycobacterium tuberculosis to first-line drugs"

Table S2 - Novel RpoB mutations.

R528C<br>N518D<br>L511P<br>Q513V<br>Q510H<br>F506L<br>I572F<br>514 L 516<br>511 P 513<br>511 E 513	(Sinha *et al.*, 2020)
512 R 514	(Hirani *et al.*, 2020)
Q172R	(Wang *et al.*, 2022)
T48A<br>Q253R<br>V259M<br>V251Y<br>V251F	(Maningi *et al.*, 2018)
G507S<br>T508A<br>L511V<br>Del 513-526<br>P520P<br>L524L<br>R528H<br>R529Q<br>S531F<br>I 572 P/F<br>E562Q<br>P564S<br>Q490Y	(Takawira *et al.*, 2017)

References

Hirani N, Joshi A, Anand S, Chowdhary A, Ganesan K, Agarwal M and Phadke N (2020) Detection of a novel mutation in the *rpoB* gene in a multidrug resistant *Mycobacterium tuberculosis* isolate using whole genome next generation sequencing. J Glob Antimicrob Resist 22:270–274.

Maningi NE, Daum LT, Rodriguez JD, Said HM, Peters RPH, Sekyere JO, Fischer GW, Chambers JP and Fouriea PB (2018) Multi- and extensively drug resistant *Mycobacterium tuberculosis* in South Africa: A molecular analysis of historical isolates. J Clin Microbiol 56:e01214-17.

Sinha P, Srivastava GN, Tripathi R, Mishra MN and Anupurba S (2020) Detection of mutations in the *rpoB* gene of rifampicin-resistant *Mycobacterium tuberculosis* strains inhibiting wild type probe hybridization in the MTBDR plus assay by DNA sequencing directly from clinical specimens. BMC Microbiol 20:284.

Takawira FT, Mandishora RSD, Dhlamini Z, Munemo E and Stray-Pedersen B (2017) Mutations in *rpoB* and katG genes of multidrug resistant *Mycobacterium tuberculosis* undetectable using genotyping diagnostic methods. Pan Afr Med J 27:145.

Wang L, Yang J, Chen L, Wang W, Yu F and Xiong H (2022) Whole-genome sequencing of *Mycobacterium tuberculosis* for prediction of drug resistance. Epidemiol Infect 150:e22.
