# Supplementary material for: Mutations and insights into the molecular mechanisms of resistance of Mycobacterium tuberculosis to first-line
Source: Genet Mol Biol. 2023 Jan 23;46(1 Suppl 2):e20220261. doi: 10.1590/1678-4685-GMB-2022-0261 (PMC9887390; doi:10.1590/1678-4685-GMB-2022-0261)
Supplement: Table S3 - [file 1415-4757-GMB-46-1-s2-e20220261-s3.pdf]

Genetics and Molecular Biology
DOI: https://doi.org/10.1590/1678-4685-GMB-2022-0261

Supplementary Material to "Mutations and insights into the molecular mechanisms of resistance of Mycobacterium tuberculosis to first-line drugs"

Table S3 - Novel PncA mutations.

Promoter ΔG(−5)<br>4 frameshift (cgTTG)<br>ΔIV(6,7)<br>Q10H<br>16 frameshift (GGgT)<br>V93M<br>122frameshift(cggCAA)<br>132frameshift(cGGT)<br>G132R<br>A146P<br>T177P	(Daum *et al.*, 2019)
L19R<br>R140H<br>E144K	(Khan *et al.*, 2019)
T167I<br>A92P<br>V125A<br>S59Y<br>L85P<br>403_413del ACCGATCATTG frameshift<br>380_381ins GG frameshift<br>50_51insC frameshift<br>21_27del CGACGTG frameshift<br>V93A<br>29_37delAGAACGACT frameshift<br>240_241insT frameshift<br>201_202insT frameshift<br>416_417insG frameshift<br>D86G<br>V180F<br>C138G<br>407_408ins TCATTGTGTGCGCCAGACGGC frameshift<br>470_471ins GG frameshift<br>500_501ins CACCGT frameshift	(Li *et al.*, 2021)

G132G (Synonymous)<br>136 delG frameshift<br>520 delG frameshift<br>S67W<br>464_465insG frameshift<br>137_138 insC frameshift<br>491_492insC frameshift<br>521_522insT frameshift<br>T142R<br>G78R<br>S104G<br>V130M	

References

Daum LT, Konstantynovska OS, Solodiankin OS, Poteiko PI, Bolotin VI, Rodriguez JD, Gerilovych AP, Chambers JP and Fischer GW (2019) Characterization of novel *Mycobacterium tuberculosis pncA* gene mutations in clinical isolates from the Ukraine. Diagn Microbiol Infect Dis 93:334–338.

Khan MT, Junaid M, Mao X, Wang Y, Hussain A, Malik SI and Wei DQ (2019) Pyrazinamide resistance and mutations L19R, R140H, and E144K in Pyrazinamidase of *Mycobacterium tuberculosis*. J Cell Biochem 120:7154–7166.

Li K, Yang Z, Gu J, Luo M, Deng J and Chen Y (2021) Characterization of *pncA* Mutations and Prediction of PZA Resistance in *Mycobacterium tuberculosis* Clinical Isolates From Chongqing, China. Front Microbiol 11:594171.
