# Supplementary material for: Mutations and insights into the molecular mechanisms of resistance of Mycobacterium tuberculosis to first-line
Source: Genet Mol Biol. 2023 Jan 23;46(1 Suppl 2):e20220261. doi: 10.1590/1678-4685-GMB-2022-0261 (PMC9887390; doi:10.1590/1678-4685-GMB-2022-0261)
Supplement: Table S4 - [file 1415-4757-GMB-46-1-s2-e20220261-s4.pdf]

Genetics and Molecular Biology
DOI: https://doi.org/10.1590/1678-4685-GMB-2022-0261

Supplementary Material to "Mutations and insights into the molecular mechanisms of resistance of Mycobacterium tuberculosis to first-line drugs"

Table S4 - Novel EmbC, EmbA, EmbB mutations.

embC D329E<br>*embA* G(−5)A<br>*embA* V18F	(Sun *et al.*, 2018)
embB S317P<br>*embB* Q445R	(Park *et al.*, 2018a)
embB Y319D<br>*embB* H1002R	(Park *et al.*, 2018b)
embB D78G	(Li *et al.*, 2017)

References

Li D, Song Y, Zhang CL, Li X, Xia X and Zhang AM (2017) Screening mutations in drug-resistant *Mycobacterium tuberculosis* strains in Yunnan, China. J Infect Public Health 10:630–636.

Park J, Jang W, Kim M, Kim Y, Shin SY, Park K, Kim MS and Shin S (2018a) Molecular drug resistance profiles of *Mycobacterium tuberculosis* from sputum specimens using ion semiconductor sequencing. J Microbiol Methods 145:1–6.

Park J, Shin SY, Kim K, Park K, Shin S and Ihm C (2018b) Determining genotypic drug resistance by ion semiconductor sequencing with the ion ampliSeq^{TM} TB panel in multidrug-resistant *Mycobacterium tuberculosis* isolates. Ann Lab Med 38:316–323.

Sun Q, Xiao TY, Liu HC, Zhao XQ, Liu ZG, Li YN, Zeng H, Zhao LL and Wan KL (2018) Mutations within *embCAB* are associated with variable level of ethambutol resistance in *Mycobacterium tuberculosis* isolates from China. Antimicrob Agents Chemother 62:e01279-17.
